# Rapid Manufacturing Method of Cardiovascular Models for Experimental Flow Analysis

**DOI:** 10.1016/j.mex.2024.103124

**Published:** 2024-12-21

**Authors:** Jarrett Fowler, Andrew B. Robbins, Cathryn Gunawan, Andrew Jastram, Michael Moreno

**Affiliations:** a Texas A&M University Department of Biomedical Engineering, College Station, TX 77840, US; b Rice University Department of Bioengineering, Houston, TX 77030, US; c University of Texas at Tyler Department of Mechanical Engineering, Tyler, TX 75799, US; d J. Mike Walker ’66 Department of Mechanical Engineering, College Station, TX 77840, US; e Texas A&M University School of Engineering Medicine, Houston, TX 77030, US; f Texas A&M University Department of Multi-Disciplinary Engineering, Houston, TX 77840, US

**Keywords:** Aorta, Manufacturing, Biomechanics, Cardiovascular, Molding, Silicone, Fabrication Method of Patient-Specific Cardiovascular Phantom Models for Experimental Fluid Flow Analysis

## Abstract

Physical anatomical models constructed from medical images are valuable research tools for evaluating patient-specific clinical circumstances. For example, 3D models replicating a patient's internal anatomy in the cardiovascular system can be used to validate Computational Fluid Dynamics (CFD) models, which can then be used to identify potential hemodynamic consequences of surgical decisions by providing insight into how blood and vascular tissue mechanics may contribute to disease progression and post-operative complications. Patient-specific models have been described in the literature; however, rapid prototyping models that achieve anatomical accuracy, optical transparency, and thin-walled compliance in a cost and time-effective approach have proven challenging. This limits their utility for modeling flows in vessels, *e.*g*.*, the aorta, where compliance is particularly important. The work described herein is focused on a unique design and fabrication process implemented to produce physical patient-specific models that replicate the original anatomy dimensions and compliance with optical properties consistent with clinical imaging techniques. The patient-specific models are produced for under $150 of easily accessible consumable raw materials within 30 h using a relatively basic approach.

• This method can be tuned for anatomies with different shapes and compliance.
• This method can produce models to investigate medical device performance *in vitro*.

## Graphical abstract

**Figure:**
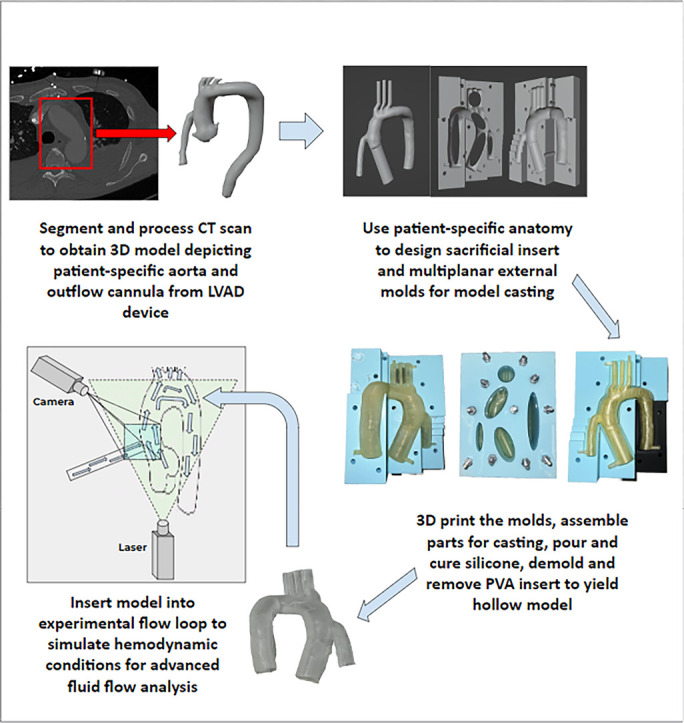

Specifications table

**Table:** 

Subject area:	Engineering
More specific subject area:	Patient-specific phantom models for experimental fluid flow analysis
Name of your method:	Fabrication Method of Patient-Specific Cardiovascular Phantom Models for Experimental Fluid Flow Analysis
Name and reference of original method:	N/A – The method was developed because there were no available solutions for creating patient-specific, thin-walled, optically clear anatomical models for more complex geometries to simulate and investigate physiological flow *in vitro.*
Resource availability:	SYLGARD 184 Silicone Elastomer PVA Filament

## Background

Cardiovascular disease is the leading cause of death in the developed world and has contributed to approximately 1 billion deaths in 2020 [1]. Hemodynamic factors play a central role in developing cardiovascular diseases [2], but studying patient-specific hemodynamics is challenging, as it requires the development of patient-specific computational models and/or patient-specific physical models. While Computational Fluid Dynamics (CFD) modeling is sometimes the preferred mode for studying patient-specific flow conditions, physical phantom models that depict patient-specific anatomy have several applications, including, but not limited to, validating a CFD models' performance [3].

In addition to CFD validation, simulating *in vivo* hemodynamics *in vitro* provides a platform for visualizing fluid flow for more in-depth mechanical investigations [4]. Current methods for observing the mechanical nature of blood and tissue *in vivo*, such as positron emission tomography (PET), functional magnetic resonance imaging (fMRI), and ultrasound, are limited with spatial and temporal resolution and functional sensitivity [5]. Particle image velocimetry (PIV) is a popular experimental imaging technique that illuminates a region for flow profile visualization to obtain highly detailed velocity and vector properties [6]. This experimental analysis offers better flow measurements than *in vivo* methods and can expand the mechanical assessment of fluid-tissue interactions, which are difficult and expensive to obtain *in silico*.

Physical anatomical models are a key component of these experimental studies and are non-trivial to fabricate depending on the anatomy and model requirements. To the authors’ knowledge, no cost-effective methods for producing complex thin-walled patient-specific physical models fit for fluid flow analysis are described in the literature. Compliant physical models of idealized anatomy have been described with good optical properties; however, the fabrication method is limited to simple geometries [7]. Another method has produced optically clear models from complex brain arteries, but the anatomy is contained within a silicone block, failing to reproduce the mechanical compliance resulting from thin-walled vessels [8].

Motivated by a study on hemodynamic-related complications of left ventricular assist devices (LVADs), a popular bridge-to-transplant and extended lifetime treatment for end-stage heart failure individuals, a patient-specific physical model is desirable to validate complex CFD simulations [9,10]. The primary objective of this method is to fabricate patient-specific, thin-walled physical models from computed tomography (CT) data that can be used to visualize fluid flow *in vitro*. The aorta model fabricated in this paper was designed for low-cost benchtop manufacturing via 3D printing and silicone casting. We have successfully utilized the model in an experimental mock circulatory loop to simulate a patient's hemodynamics with an implanted LVAD. The model's functionality under physiological flow while displaying imaging properties is briefly summarized to exhibit its practicality and potential for tangential applications.

## Method Details

**Fig. 1:**
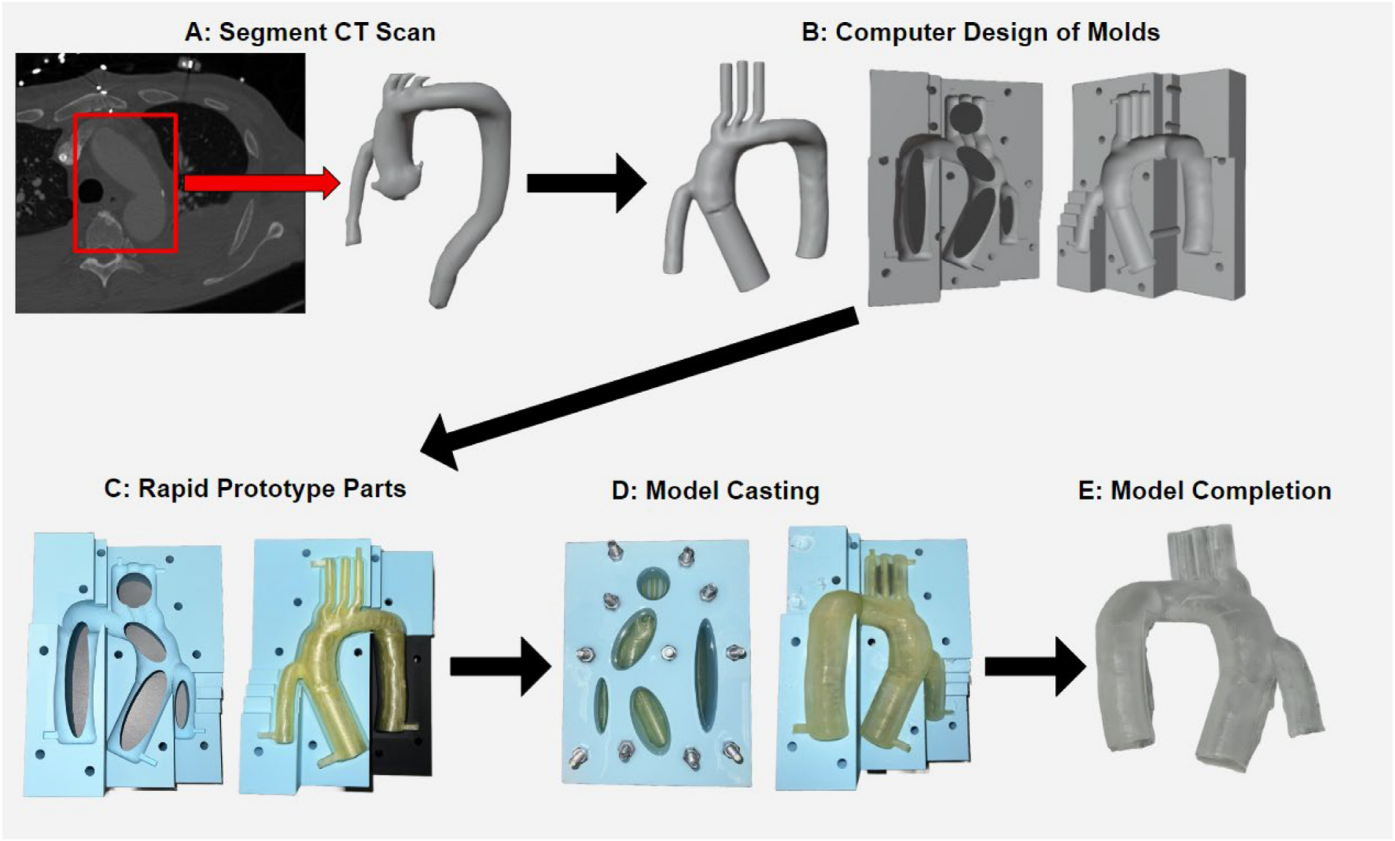
Manufacturing process for physical models. A) Processing Medical Imaging Data. The CT scan was segmented to isolate and export the aorta and outflow cannula as a 3D object. B) Designing the Molds Using Computer-Aided Design. The 3D anatomical object was utilized to create the sacrificial insert with extended segments, which was used to create multiplanar external molds. C) Producing Molds Using 3D Printing. The insert and external molds were 3D printed and assembled for the casting procedure. D) Casting the Model Out of Silicone. An optically clear silicone was poured into the assembled mold and cured to create a thin-walled silicone model. E) Post-Casting Model Processing. The sacrificial insert is removed to yield a hollow, optically clear, thin-walled aorta model.

The following method details the fabrication of anatomical models for experimental use by processing medical imaging data to design internal and external molds for a traditional casting procedure. The design of the external molds is critical to obtaining a thin-walled model, and the multiplanar approach expanded upon in subsequent paragraphs is optimal for manufacturing complex geometries. Before casting the model via multi-planar molds, the group experimented with rotational mold casting and directly resin printing the model. For rotational casting, a fused deposition modeling (FDM) 3D printed mold containing a cavity of the aorta geometry was filled with silicone and rotated randomly, coating the interior with silicone to produce a hollow model. This was limited by the inability to tune model thickness in different sections, resulting in the pooling of narrow regions, such as the outflow cannula angle. We presumed that directly resin printing the model with less post-processing than silicone casting was a more convenient option; however, the resin prints lacked accuracy for the complex models with susceptibility to warping. Resin printing can also be explored to expedite fabricating the molds for casting, but the molds would need surface treatment as most resins inhibit silicone curing. Thus, the method herein was the most effective and convenient route for producing a model fit for flow visualization while providing tunable metrics for broad applications. Fig. 1 displays a flowchart of the manufacturing process and the simplicity of the method.

## Processing CT Data

One CT scan of a deidentified LVAD patient was received from collaborators at the Houston Methodist Hospital in Houston, Texas, as part of a study investigating complications related to the relationship between the inflow and outflow cannula angulation [11]. The CT scan depicts the patient-specific blood volume of the aortic arch with the outflow cannula from the LVAD into the ascending aorta. The CT scan is segmented in Slicer (Slicer.org) to obtain a 3D model that can be exported as a stereolithography (STL) file. For this application, the region of interest (ROI) is the ascending aorta, aortic arch, base of left and right subclavian and carotid arteries, descending aorta, and outflow cannula from the LVAD. Within the Slicer software, the aorta and inflow cannula can be segmented as separate objects to process each anatomical feature efficiently. The ‘islands’ and ‘scissors’ tools are utilized to remove extraneous objects surrounding each segment, and the ‘smoothing’ tool helps solidify the object by removing holes under the specified kernel size. Images of the CT scan and resulting 3D object are shown in Fig. 1**A.** Once the outflow cannula and aorta are segmented and processed, the ‘logical operators’ tool combines the two segments to create one object. The final 3D model is exported as an STL file for subsequent modeling to make the molds necessary for casting.

## Mold Modeling

The STL file containing a 3D model depicting the patient-specific aortic arch and outflow cannula is imported into Blender 3D Modeling and Animation software (Blender.org) for model refinement and to create the exterior mold and sacrificial insert. The 3D model is first scaled down to 0.7x the original size to improve the model casting process and consume less materials during process development. It is worth noting that the mold design accommodates objects of different sizes depending on the desired features and applications. The scaled aorta contains the base of the aortic heart valve, brachiocephalic artery, left subclavian artery, and left carotid artery, which will all be extended for insertion into the mock circulatory loop. Cylindrical mesh objects that match the diameter of each artery are aligned and connected to the aortic model using the ‘boolean union’ tool (Fig. 1**B**). Now, with extended segments, the model comprises the internal sacrificial anatomy and will be used to design the two external molds.

**Fig. 2:**
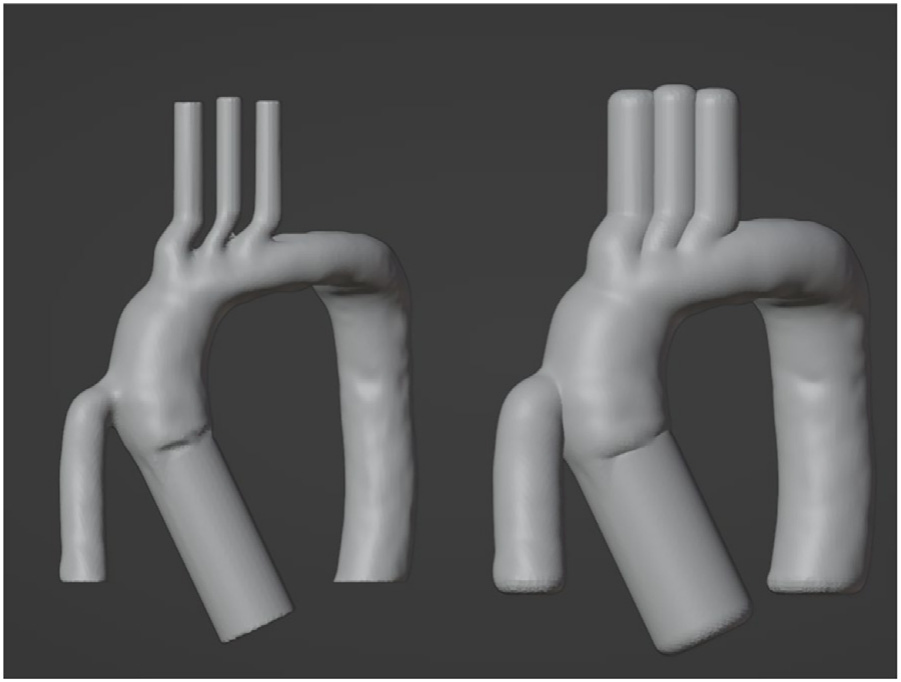
Create 3–4 mm offset. The insert aorta (left) is uniformly scaled 3.5 mm to get the offset aorta (right), which will make the cavity.

**Fig. 3:**
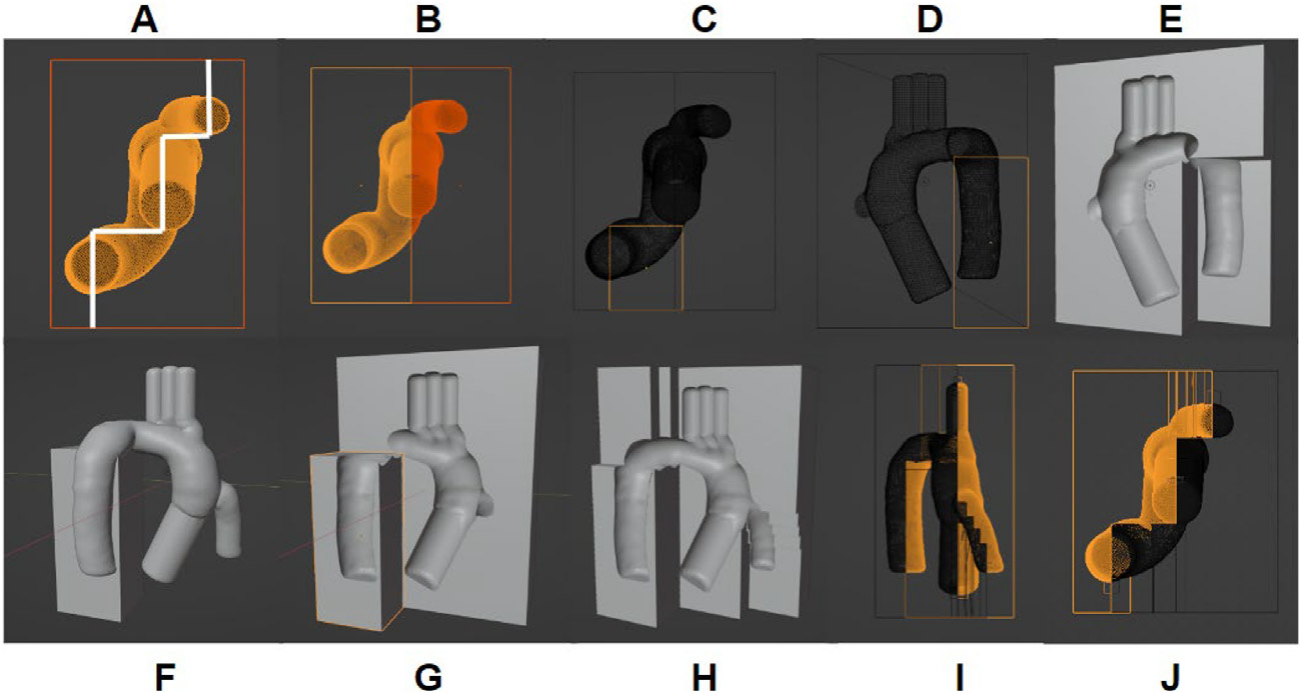
External Mold Design. A) Mold Outline. The schematic for evenly splitting the non-symmetric model. B) Two External Molds. Subtracting the offset aorta from two rectangle meshes. C, D) Isolate Segments. Create a rectangular mesh for each segment of the anatomy. E) Negative Extrusion. Subtract the rectangular mesh from the external mold enclosing the segment. F) Positive Extrusion Part A. Subtract the offset aorta from the rectangular mesh. G) Positive Extrusion Part B. Add the rectangular mesh with the aorta cavity to the external mold missing the segment. H) Repeat A-G. Perform each segment's positive and negative extrusion until the entire anatomy is evenly split between the external molds. I) Side-View of Multiplanar Mold. The two molds evenly split each segment. J) Bottom -View of Multiplanar Mold. Each segment of the aorta is split between the external molds, completing the schematic outlined in A.

The cavity of the external models needs to be offset from the sacrificial insert to create the vessel wall, as the 3D model depicting the insert represents the inner blood volume. The ‘shrink/flatten’ tool in Edit Mode enables a uniform scaling of the object that maintains angular relationships, a necessary feature for the insert to be centered in the cavity (Fig. 2). Aorta dimensions vary depending on the demographic, but the wall thickness of adult aortas is typically between 3 and 4 mm [12], which will be the offset value for the external cavity. Once the anatomical geometry is offset, the model is subtracted from two equal cube meshes to create the external molds. The novelty of this approach lies in designing the external molds for adaptation to a model with extruding segments in different directions. Typically, model casting is performed with two external molds that split the object via one plane; however, this feat is difficult when the geometry making up the cavity does not lie evenly along one plane. To address this challenge with the aorta, each external half will be designed to evenly split the various segments of the model, creating two multi-planar molds. This concept is visualized in Fig. 3**A**, where the aorta model lies along multiple planes, and each half of the mold has varying extrusion levels to split the inner model. The external mold design begins with placing the offset aorta geometry between two cube meshes (55 mm x 130 mm x 170 mm) and visualizing where each segment will be split (Fig. 3**B**). A series of ‘boolean union’ and ‘boolean difference’ modifications are performed to design the multi-planar molds, beginning with subtracting the aorta from each block. Next, smaller cube meshes are designed to evenly split each segment of the model (Fig. 3**C**). The offset aorta is subtracted from the cube, which is then combined and subtracted from the respective external half, creating an extruded segment of the mold that evenly splits the anatomical feature (Fig. 3**E-G**). This process is repeated until the cavity is evenly split between the external molds (Fig. 3**H**). Future iterations of this process are to incorporate curved contours that follow the profile instead of flat steps.

**Fig. 4:**
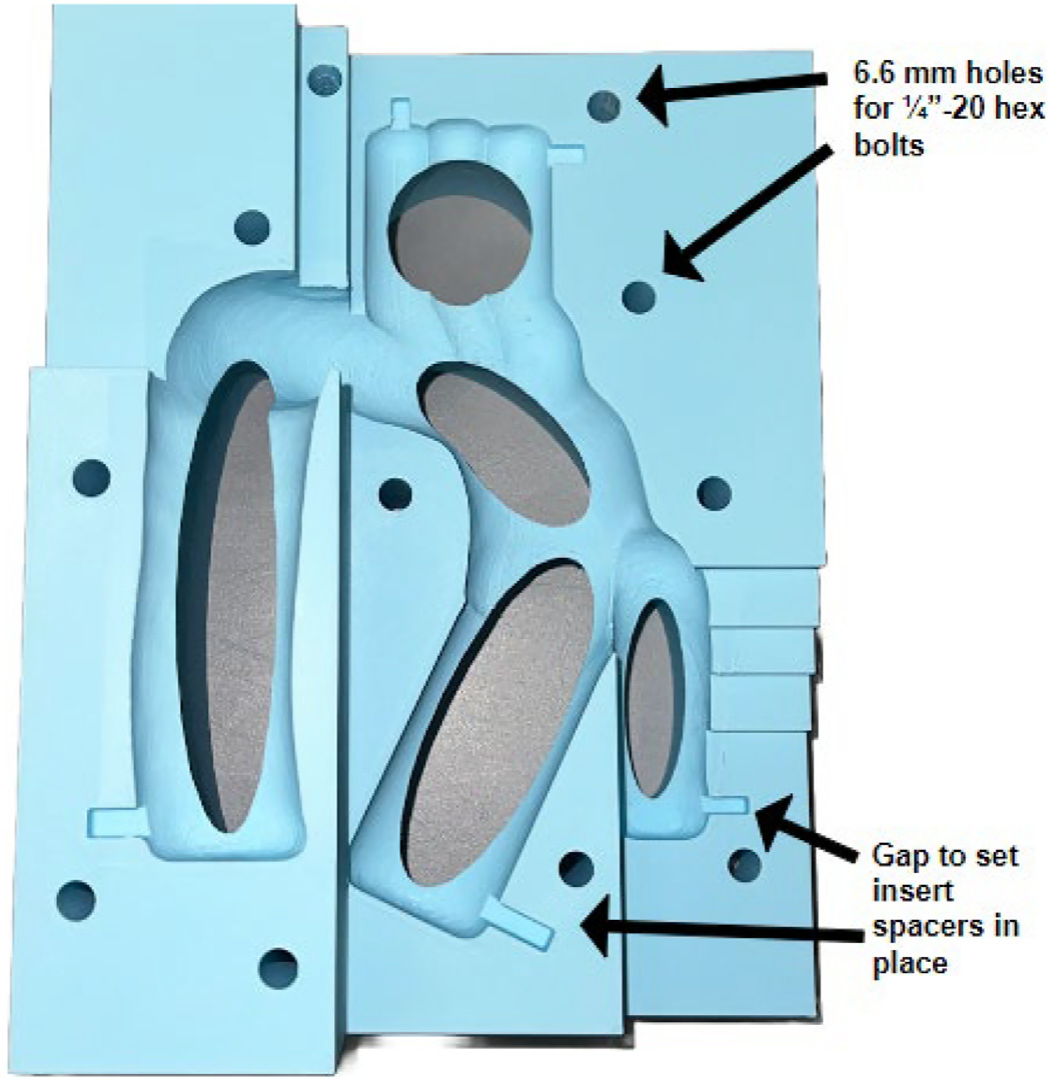
External Mold Diagram with Improved Features.

Small features like spacers, vents, and bolt holes were added throughout different iterations to improve the casting procedure (Fig. 4). We found spacers necessary for evenly suspending the internal model within the cavity to achieve a uniform wall thickness. Rectangle meshes were added to the ends of each extruded segment of the internal mold and subtracted from the external molds to insert the internal mold firmly. The rectangular spacers need to be sized up 0.1 mm in all directions before being removed from the external mold, as we found the physical dimensions to vary within 0.05–0.1 mm of the computer models. The last step of the mold design is the incorporation of vents for pouring silicone and bolt holes to fasten the external molds together. The vent design outlines the anatomical model and is created by subtracting cylindrical meshes from one external half. Throughout the iteration process, we found that wider vents provide more accessibility to remove bubbles formed from pouring silicone. Holes were added to both external molds by subtracting cylindrical meshes with 6.6 mm diameter, suitable for 1/4″−20 bolts.

## Rapid Prototyping of Molds

**Fig. 5:**
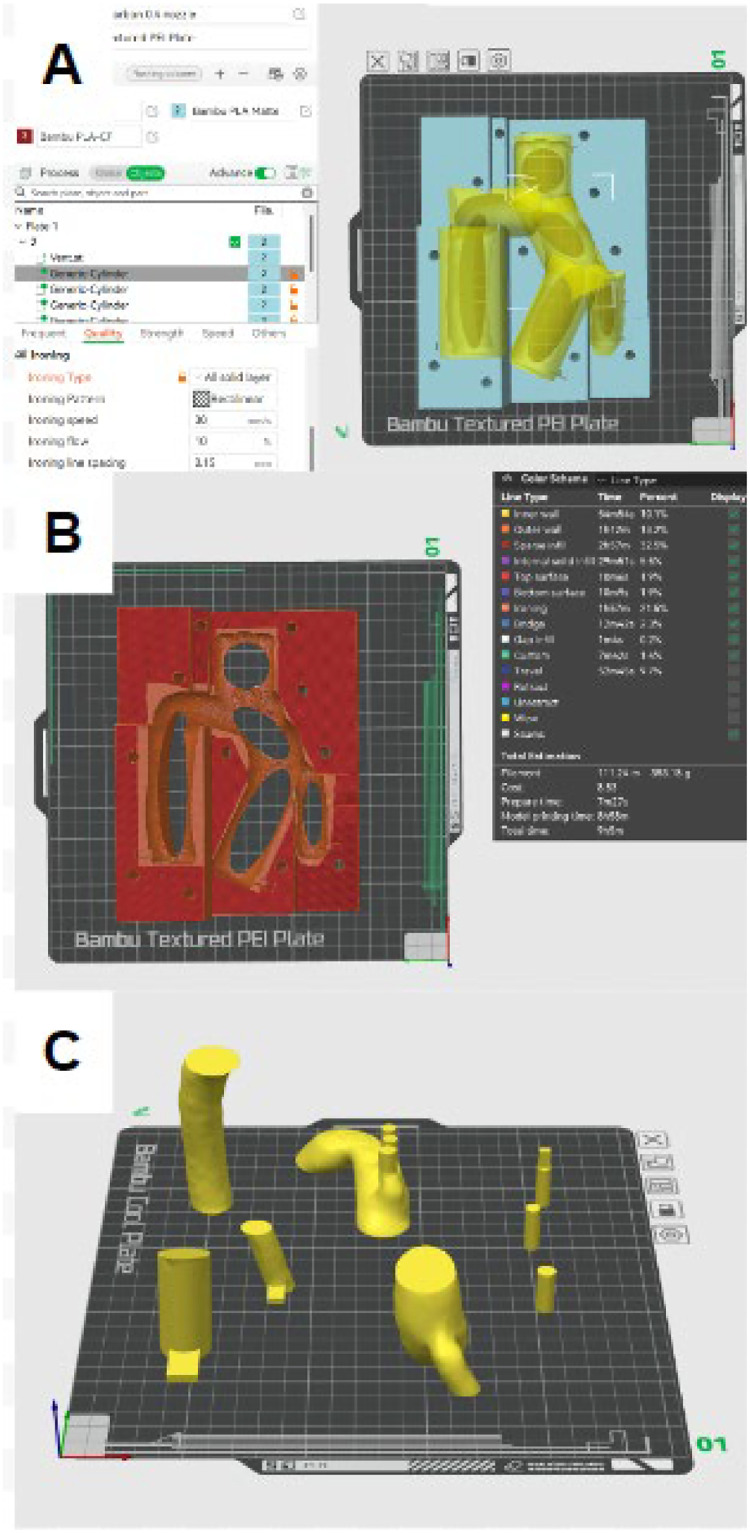
Print Setting for the External Molds (A,B) and Sacrificial Insert (C). A) Cylindrical modifiers added to the part to improve surface quality via ironing. B) Visual effects of ironing modifiers on the surface after slicing the external mold. C) The sacrificial insert was dissembled for the improved printing process.

The sacrificial insert and external molds are 3D printed with a Bambu Lab X1-Carbon Combo 3D printer (Bambu Lab, Austin, TX) and subsequent Bambu Lab slicing software. The internal mold is printed with polyvinyl alcohol (PVA) filament (FUSED MATERIALS, New York, NY), and the external molds with a polylactic acid matte (PLA-matte) filament (Bambu Lab, Austin, TX). The filament selection can be customized depending on the application, as acrylonitrile butadiene styrene is also suitable for printing low-cost and high-quality molds. To improve the surface quality of the external molds, modifiers were placed along the cavity of the external mold to utilize the ‘ironing’ feature in the Bambu Lab slicing software with a rectilinear pattern, 30 mm/s ironing speed, 10 % ironing flow, 0.15 mm ironing line spacing, and the top surface ironing type (Fig. 5**A, B**). Due to the complex shape of the sacrificial insert, each model segment is cut and printed separately to be reassembled post-printing (Fig. 5**C**). Reassembling the sacrificial insert is relatively straightforward, using generic glue to attach the respective pieces. Additionally, PVA is a heat-sensitive filament, and the maximum nozzle temperature needs to be reduced to 220 °C. A Bambu Lab Textured PEI Plate for the PLA matte molds and a Bambu Lab Cool Plate for the PVA mold resulted in the best printing conditions for the respective molds.

## Model Casting

The casting procedure is performed after printing and preparing the sacrificial insert and external molds. First, the insert is placed into the base external mold using the spacers to firmly set the model within the cavity (Fig. 1**C**). Once the insert is secured, the vent external mold is affixed to the base external mold, lining up the varying extrusions and securing the spacers of the internal mold. The external molds are clamped shut by fastening ¼”−20 bolts through each mold to minimize silicone leaking out of the cavity. Further, we found the use of duct tape wrapped around the crease between each external mold to effectively eliminate any silicone leakage. A Dow Corning Sylgard 184 silicone is used to create the models as the material exhibits high optical transparency and tunable compliance, fitting for fabricating vessels for fluid flow visualization [13,14]. The silicone elastomer comes in a base (part A) and curing agent (part B), mixed with a 10:1 wt ratio base to curing agent. Filling up this mold, including space in the vents, consumes 322.25 gs of part A and 32.23 gs of part B, mixed in three separate cups. Mixing the silicone in multiple containers accelerates the degassing process, which occurs after mixing and before pouring to eliminate bubble formation. The silicone is placed in a vacuum chamber and held at 27 in-Hg of vacuum until the solution is visually bubble-free. Once properly mixed and degassed, the silicone is distributed throughout the mold. Bubbles have the potential to form while pouring, so careful, slow deposition of the silicone into one segment at a time, allowing bubbles to rise to the surface, will result in the highest-quality model. Once the mold is filled to the brim, set aside for approximately 30–45 min to allow any additional bubble formation to rise before placing the construct in an oven at 60 °C for accelerated curing.

After about 8 h, the construct is removed to begin demolding. The duct tape is removed, bolts unfastened, and two external molds are pried open using a flat-head driver, being careful not to damage the cured silicone. The silicone insert is firmly set in the vent external mold due to the cured silicone throughout the vents. We found removing the insert to be quite tedious, but multiple tactics proved helpful. First, removing silicone from the vents helped release the insert. Extreme care should be taken so as not to damage the model or tear the silicone; the difficulty will depend on the complexity of the geometry. Once the cured model is removed from both external molds, the ends of each segment are entirely removed to expose the PVA insert. The PVA mold is removed using high-temperature water and a soft scraper. The dissolving process takes approximately one hour to completely remove the PVA, resulting in a hollow, optically clear, thin-walled aorta model (Fig. 1**E**).

## Method Validation

The purpose of this work is to demonstrate a hollow, optically clear, thin-walled, patient-specific physical model for experimental fluid flow analysis applications. The final method is a culmination of a lengthy iteration process that evaluated numerous techniques and approaches until a fabricated model exhibited anatomical accuracy, physiological compliance, and optical transparency. Our defined success threshold for each criterion was replicating 95 % of the original anatomy's dimensional and volume, supporting physiological flow and pressure values, and minimizing image path distortion. We also evaluated the method for cost and time efficiency to prove the convenience of this in-house method over commercial options.

## Rapid Prototyping

The manufacturing process for the aorta model takes approximately 28 h (∼6 h of computer design, 12 h of 3D printing, and 10 h for the casting and curing process) with <3 h of active labor time. The raw materials used to fabricate the model described in this paper amount to roughly $130 (85 % for the silicone and 15 % for the filament). Additionally, the fabrication method has produced five models at 70 % and 80 % (bigger models had longer print times and consumed more materials) of the original CT scan, proving the method's reproducibility. The current multiplanar iteration has a 100 % success rate with the past five models, compared to a ∼20 % success rate of previous mold designs.

## Anatomical Accuracy

**Fig. 6:**
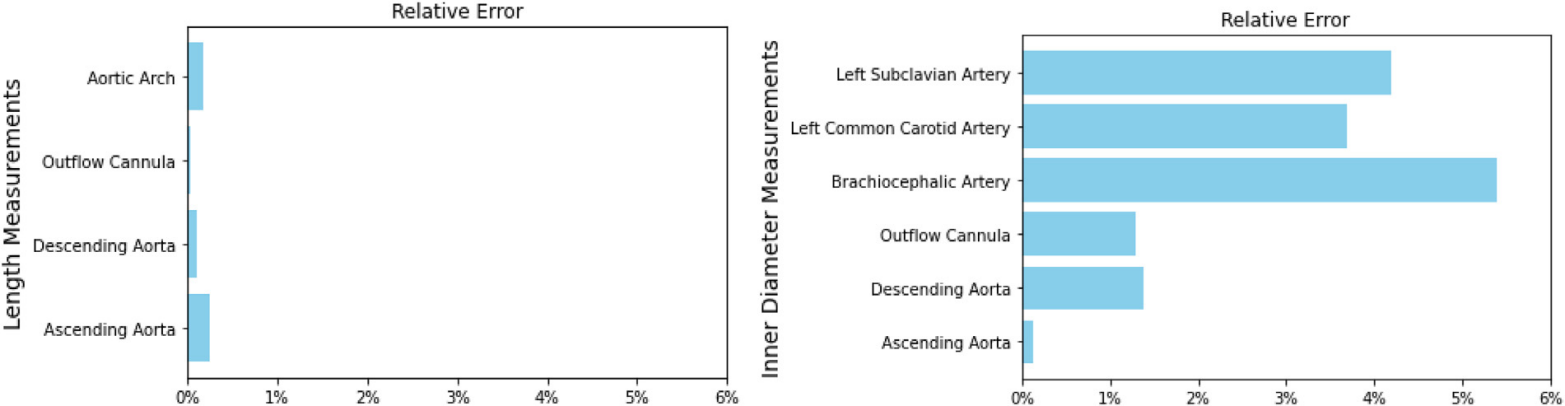
Dimensional Comparison. Left) Length Comparison. The maximum length relative error is 0.238, exceeding the 95 % success threshold. Right) Inner Diameter Comparison. The inner diameter values had higher variation in the brachiocephalic and left common carotid and subclavian arteries.

The inner diameter of each extended segment and the length of anatomical segments were measured for the physical and CT models. Fig. 6 displays the percent difference in the length (**Left**) and inner diameter (**Right**) between the two models, validating that the physical model falls within 95 % of the original dimensions. The average wall thickness of the physical model is 2.988 ± 0.692 mm, compared to the cavity's 3.5 mm design. More minor diameter features are more likely to move during the casting process, and the spacers' design can be optimized to improve thickness distribution and dimensional accuracy.

In addition to the dimensional evaluation, CT scans of the physical model were taken to obtain a digitalized model of the physical blood volume for a volume analysis. The volumes, excluding the cylindrical extensions added to the model for inflow and outflow purposes, were compared using Blender. The physical model is within 96.7 % of the original volume, indicating a 0.989 mm/mm linear shrinkage. A volumetric decrease of 3 % is consistent with the shrinkage of Sylgard 184 after cooling from a 60 °C curing temperature to room temperature (∼22 °C) and could be corrected for by adjusting the dimensions to account for shrinkage or curing the model at room temperature [15]. However, the minor shrinkage did not impede this application's functionality.

## Mechanical Compliance

**Fig. 7:**
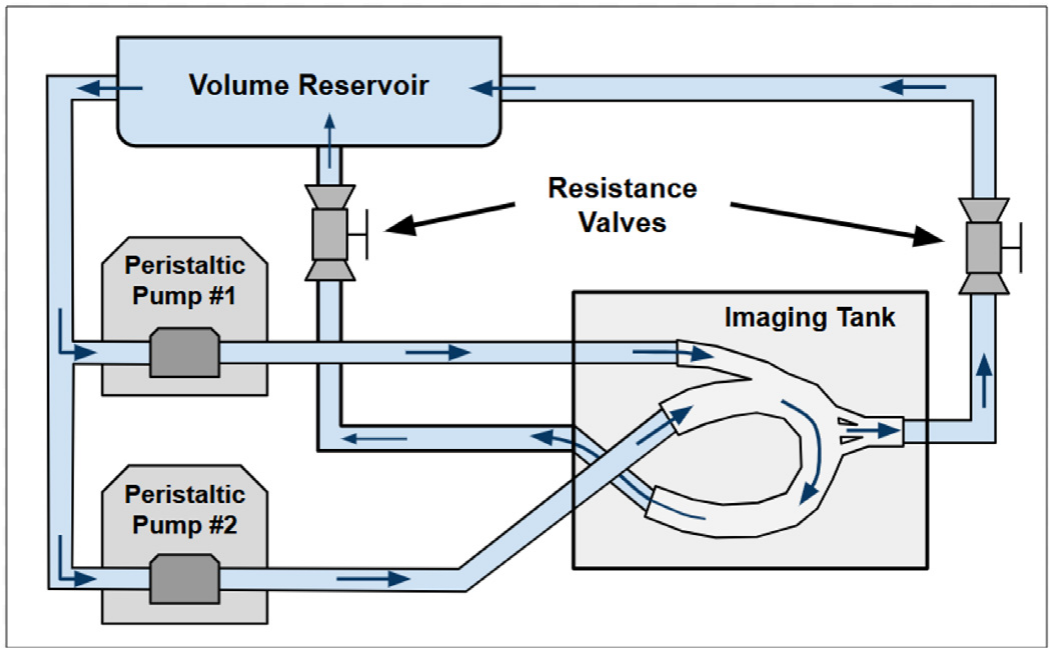
Mock circulatory loop schematic. The custom flow loop for the LVAD aorta model simulates cardiac output and continuous LVAD flow with tuned peripheral resistance.

**Fig. 8:**
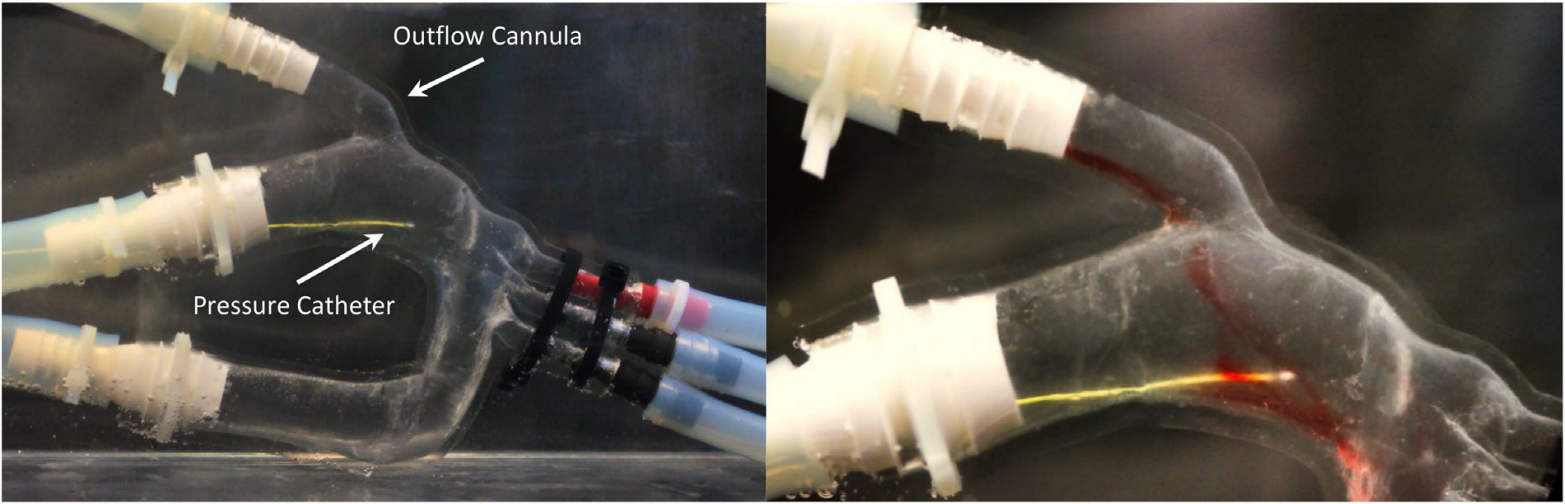
Flow Profile Visualization. Left) The model was inserted into the imaging tank to minimize image path distortion. Right) Red dye was injected into the flow loop to visualize physiological flow through the outflow cannula into the ascending aorta.

The model's compliance was evaluated by observing functionality under physiological flow and pressure when inserted into an in-house mock circulatory loop (Fig. 7). The flow loop utilizes resistance valves and oscillating flow to simulate 60 bpm, a maximum flow rate of 5.5 L/min, and a maximum pressure of 140 mm Hg, the peak systolic pressure experienced in the aorta [16,17]. The model's capacity to maintain function under physiological parameters validates the mechanical integrity of the thin-walled silicone and proves helpful for investigations requiring accurate anatomical compliance. Fig. 8 depicts the flow profile through the outflow cannula of the model inserted into the imaging tank within the flow loop.

## Refractive Index Matching

**Fig. 9:**
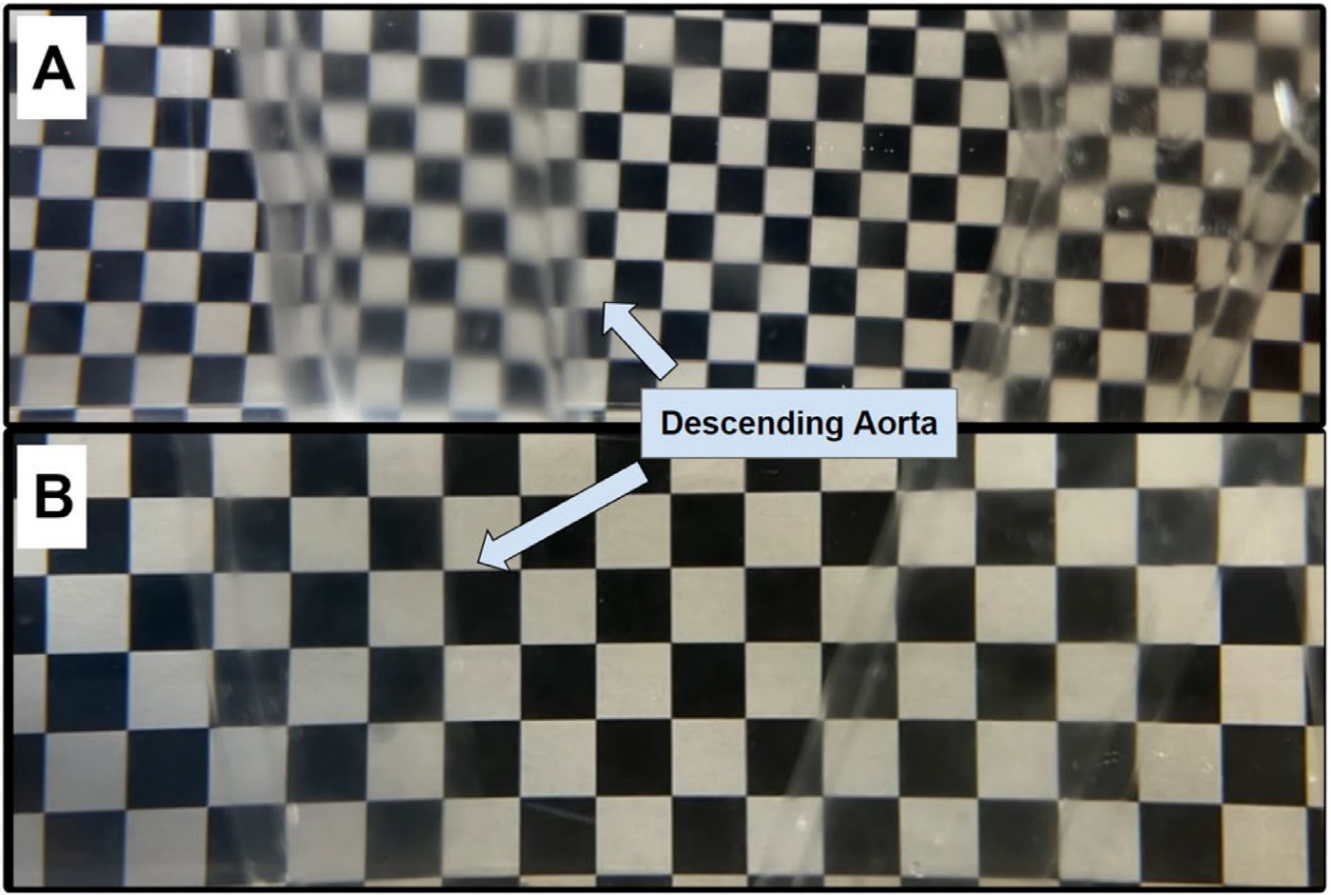
Refractive Index Matching with 60:40 Water-Glycerol and Urea. A) Distorted Model. The silicone model is noticeably more visible and distorts the gridlines in a solution with a refractive index of 1.40. B) Transparent Model. The silicone model submerged in a solution with a refractive index of 1.42 is practically transparent, resulting in minimal distortion of the gridlines.

The imaging tank outlined in the schematic (Fig. 7) contains a solution with a matched refractive index to render the model transparent. The solution consists of a 60:40 water-glycerol mixture, a standard blood-analog fluid with similar density and viscosity to blood, and urea, a mineral that alters the refractive index without significantly changing the fluid mechanical properties [18]. Sylgard 184 has a registered refractive index that varies between 1.40 and 1.44, depending on curing conditions. Whereas a 60:40 water-glycerol solution has a refractive index of 1.39. Thus, we incrementally added urea until the model displayed no visible image path distortion. The exact distortion was evaluated by placing a checkered gridline behind one wall of the model, as the goal is to visualize flow through one wall of the model with minimal image path distortion. For example, Fig. 9**A** displays high gridline distortion, hindering imaging capabilities. Comparatively, Fig. 9**B** shows an almost entirely transparent model with submersion in a 60:40 water-glycerol solution containing 26.13 wt % urea. A digital Cole Parmer refractometer (Model 81,150–55) registered a refractive index of 1.42 for the solution, falling within the expected range of Sylgard 184.

This method can be customized for different anatomies, designing a multiplanar mold fit for the specific geometry. Depending on the application, changes in the materials may also be necessary, such as using a more robust silicone. While some design iterations may be required, this molding method can produce complex thin-walled geometries with many desired characteristics in a wide range of experimental fluid flow applications.

## Limitations and Discussion

While the method detailed in this paper is relatively straightforward, there are potential limitations. The manufacturing timeline can vary depending on the complexity of the mold design. Additionally, the method is adaptable for different anatomies, but the complexity of the geometry could potentiate more cumbersome mold design and casting success. The silicone used in this project resulted in geometry and wall thickness limitations due to the low tear resistance, with increased susceptibility of silicone tearing for models with a wall thickness below 1 mm. Alternative materials can be explored to circumvent these challenges and optimize model properties. The silicone materials Clear Flex™ and SORTA-Clear™ potentiate adequate casting materials for models with higher tear resistance, achieving models with a wall thickness below 1 mm that withstand de-molding and support fluid flow. However, SORTA-Clear™ lacks the optical transparency necessary for imaging, and both Clear Flex™ and SORTA-Clear™ have high viscosity that complicates the casting process due to the difficulty of removing bubbles. Casting a model with a more viscous silicone can potentially improve mechanical robustness. Still, the mold design and casting procedure need further optimization, specifically, the mold's vents and orientation, to avoid and remove bubble formation during the pouring process. The Sylgard 184 silicone elastomer is favorable due to its tunable mechanical properties and imaging capabilities, which, matched with the multi-planar casting approach, offers a highly customizable method for producing models of varying geometries and anatomical features.

## CRediT Author Statement

**Jarrett Fowler:** Conceptualization, Methodology, Software, Validation, Formal analysis, Investigation, Resources, Data Curation, Writing – original draft, Writing – review & editing, Visualization. **Andrew Jastram:** Conceptualization, Supervision, Writing – review & editing, Project Administration. **Andrew Robbins:** Conceptualization, Supervision, Writing – review & editing, Project administration. **Cathryn Gunawan:** Data curation, Software, Validation. **Michael Moreno:** Writing – review & editing, Project administration, Funding acquisition.

## Declaration of Competing Interest

The authors declare that they have no known competing financial interests or personal relationships that could have appeared to influence the work reported in this paper.

## Data Availability

Data will be made available on request.
